# Supplementary material for: Bloodletting for Acute Stroke Recovery: A Systematic Review and Meta-Analysis
Source: Healthcare (Basel). 2024 Oct 17;12(20):2060. doi: 10.3390/healthcare12202060 (PMC11507497; doi:10.3390/healthcare12202060)
Supplement: Supplementary file 1 [file healthcare-12-02060-s001.zip › Table S2. Search strategy.Embase수정.pdf]

Table S2. Search strategy

Search time	from inception to 4 June 2024
**PubMed**	(((((((stroke[Title/Abstract]) OR (cerebral infarction[Title/Abstract])) OR (cerebral hemorrhage[Title/Abstract])) OR (cerebrovascular disorder[Title/Abstract])) OR (cerebrovascular accident[Title/Abstract])) OR (apoplexy[Title/Abstract])) OR (brain ischemia[Title/Abstract])) AND ((((bloodletting[Title/Abstract]) OR (blood pricking[Title/Abstract])) OR (collateral pricking[Title/Abstract])) OR (fang xue[Title/Abstract]))
**Embase**	('stroke'/exp OR stroke OR 'cerebral infarction'/exp OR 'cerebral infarction' OR 'cerebral hemorrhage'/exp OR 'cerebral hemorrhage' OR 'cerebrovascular disorder'/exp OR 'cerebrovascular disorder' OR 'cerebrovascular accident'/exp OR 'cerebrovascular accident' OR 'apoplexy'/exp OR apoplexy OR 'brain ischemia'/exp OR 'brain ischemia') AND ('bloodletting'/exp OR bloodletting OR 'blood pricking' OR 'collateral pricking' OR 'fang xue' OR bloodletting:ti,ab,kw OR 'blood pricking':ti,ab,kw OR 'collateral pricking':ti,ab,kw OR 'fang xue':ti,ab,kw) AND ('clinical article'/de OR 'clinical trial'/de OR 'clinical trial topic'/de OR 'comparative effectiveness'/de OR 'comparative study'/de OR 'controlled clinical trial'/de OR 'controlled study'/de OR 'human'/de OR 'major clinical study'/de OR 'randomized controlled trial'/de OR 'randomized controlled trial topic'/de) AND ('cerebrovascular accident'/de OR 'neurologic disease'/de)
**Cochrane Library (TRIALS)**	(stroke[ti,ab,kw] OR cerebral infarction[ti,ab,kw] OR cerebral hemorrhage[ti,ab,kw] OR cerebrovascular disorder[ti,ab,kw] OR cerebrovascular accident[ti,ab,kw] OR apoplexy[ti,ab,kw] OR brain ischemia[ti,ab,kw]) AND (bloodletting[ti,ab,kw] OR blood pricking[ti,ab,kw] OR collateral pricking[ti,ab,kw] OR fang xue)
**China National Knowledge Infrastructure**	(中风[Tka] OR 卒中[Tka] OR 脑卒中[Tka] OR 脑梗死[Tka] OR 脑栓塞[Tka] OR 脑溢血[Tka] OR 脑出血[Tka] OR 缺血性脑卒中[Tka] OR 出血性脑卒中[Tka]) AND (刺络 [Tka] OR 刺血 [Tka] OR 泻血 中[Tka] OR 放血[Tka]) AND (系统[Tka] OR 随机[Tka])
**Korean language databases**	(뇌졸중 OR 뇌경색 OR 뇌출혈 OR 중풍 OR 뇌혈관질환) AND (사혈 OR 자락 OR 방혈)

**International Clinical Trials Registry Platform & Clinical Trials.gov**	Bloodletting OR blood pricking OR collateral pricking OR fang xue
**Chinese Clinical Trial Register**	刺络 [Tka] OR 刺血 [Tka] OR 泻血 中[Tka] OR 放血 OR Bloodletting OR blood pricking OR collateral pricking OR fang xue
**Clinical Research Information Service**	사혈 OR 자락 OR 방혈 OR Bloodletting OR blood pricking OR collateral pricking OR fang xue
