# Supplementary material for: Bloodletting for Acute Stroke Recovery: A Systematic Review and Meta-Analysis
Source: Healthcare (Basel). 2024 Oct 17;12(20):2060. doi: 10.3390/healthcare12202060 (PMC11507497; doi:10.3390/healthcare12202060)
Supplement: Supplementary file 1 [file healthcare-12-02060-s001.zip › Table S3. List of excluded studies.pdf]

Table S3. List of excluded studies.

First author	Year	Title	Journal	Volume	Issue	page	Reasons for exclusion
徐莹	2017	金津玉液点刺放血治疗脑卒中后构音障碍的疗效观察	智慧健康	3	24	70-71	
何铭锋	2021	刺络四缝穴治疗脑卒中后手指屈曲拘挛的临床研究	神经损伤与功能重建	16	9	547-549	
卫星	2011	刺络结合针刺对脑梗死患者颈动脉粥样硬化的影响	中医药临床杂志	23	12	1047-1050	
范小英	2014	点刺放血配合康复训练治疗脑梗死后饮水呛咳临床研究	上海针灸杂志	33	11	986-987	
杨丹	2016	井穴放血疗法对卒中后抑郁患者抑郁状况和血清5-羟色胺水平的影响研究	中国全科医学	19	4	486-488	
杨丹	2015	井穴刺络放血对脑卒中后抑郁的疗效观察	辽宁中医杂志	42	1	157-158	
纪昌春	2012	井穴点刺放血配合西药治疗脑卒中后肩手综合征疗效观察	陕西中医	33	4	471-473	
梁慧	2015	中西医结合治疗脑卒中后抑郁20例临床观察	甘肃中医学院学报	32	2	63-66	
代飞	2021	通督调神针法联合经筋结点放血治疗脑卒中后痉挛性瘫痪30例	安徽中医药大学学报	40	4	60-63	
唐术平	2015	从络病论治脑卒中后肢体麻木的临床观察	中国实用医药	10	5	268-270	
季兴	2011	哑门刺血结合语言康复治疗脑梗死单纯性运动性失语	中国针灸	31	11	979-982	
徐贞杰	2014	放血疗法配合肌注腺苷钴胺治疗脑卒中后肢体麻木临床观察	上海针灸杂志	33	1	11-13	
刘慧影	2015	针刺放血治疗对脑梗死患者血浆内皮素、凝血系统的影响	中国民康医学	27	16	78-79	
彭华	2014	针刺配合十宣放血治疗卒中手指功能障碍疗效观察	实用中医药杂志	30	2	142	
戴晓矞	2009	针刺配合十宣放血治疗卒中后肩手综合征疗效观察	四川中医	27	7	113-114	
陈幸生	2012	针刺配合刺血治疗卒中后肩手综合征疗效观察	中医药临床杂志	24	11	1076-1077	
俞红五	2014	风府穴刺血治疗脑梗死后运动性失语症临床研究	光明中医	29	6	1248-1249	
王舰	2015	风府穴刺络放血治疗脑梗死后口腔期吞咽障碍疗效观察	辽宁中医杂志	42	4	850-851	
林万庆	2021	放血疗法对脑卒中后痉挛性腕背伸功能障碍患者的影响	康复学报	31	6	461-467	No acute stage patients
施栋	2016	舌底放血配合言语康复训练治疗假性延髓麻痹引起的构音障碍	中外医学研究	14	35	50-51	
张莉君	2023	舌下刺络放血疗法联合针刺治疗中风后失语的临床疗效观察	中医药学报	51	3	85-88	
郭娜	2021	手十二井刺络放血配合拮抗肌推拿对中风患者认知和血液动力学的影响	辽宁中医杂志	48	4	180-183	
梁慧	2013	手十二井穴放血为主治疗脑卒中后抑郁状态临床观察	上海针灸杂志	32	6	457-458	
郑勇文	2018	手十二井穴放血疗法辅助治疗出血性卒中30例	广西中医药大学学报	21	2	31-32	
张斯雅	2023	手十二井穴刺络放血治疗中风后肩手综合征临床观察	云南中医中药杂志	44	1	65-68	
华晓琼	2023	循经放血疗法治疗卒中后风痰瘀阻证上肢感觉障碍的临床研究	现代中医临床	30	1	38-42	
石会	2019	循经井穴放血治疗丘脑卒中所致麻木的临床研究	中西医结合心脑血管病杂志	17	4	496-499	
石会	2017	循经井穴放血疗法治疗丘脑中风后遗症的临床疗效及其对患者血清TNF-α、IL-6和IGF-Ⅱ水平的影响	河北中医药学报	32	3	39-41	
张继庆	2007	十宣放血治疗中风后手指功能障碍60例	针灸临床杂志	23	3	30-31	
李淑英	2013	十宣放血结合针刺治疗缺血性脑卒中急性期30例	中医外治杂志	22	1	44-45	
高竹颖	2016	十宣刺血治疗中风恢复期手指麻木	中国临床研究	8	1	37-38	
王晓君	2023	功能训练结合手十二井穴刺络放血治疗中风偏瘫肢体水肿疗效观察	实用中医药杂志	39	10	2046-2047	
林舒鸿	2017	十宣穴点刺放血治疗脑梗死后手指拘挛状态患者的临床效果	医疗装备	30	15	124-125	
贾东佩	2018	十宣穴点刺放血对脑梗死患者术后神经功能及血清胱抑素C水平的影响	上海针灸杂志	37	6	609-613	
梁慧	2014	十二井穴刺络放血对中风后抑郁状态的影响	广西中医药	37	1	52-54	
钟伟华	2018	十二井穴刺络放血疗法配合康复训练对脑卒中后肩手综合征患者功能恢复	现代中西医结合	27	25	2819-2822	

		及血液流变学的影响	杂志				
乔秀颀	2022	十二井穴刺络放血疗法联合疏血通脉汤治疗脑卒中后肩手综合征临床研究	四川中医	40	12	190-192	
顾建丽	2023	耳部全息铜砭刮痧联合耳尖放血治疗脑卒中后肠道热结型便秘患者的效果观察	实用临床医药杂志	27	2	97-100	
王身林	2020	耳尖、十宣放血对高血压脑出血微创血肿清除术后患者神经功能恢复的影响	上海针灸杂志	39	11	1391-1395	
徐君仪	2020	耳尖放血治疗高血压病合并脑出血恢复期的疗效观察	中国临床研究	12	8	42-44	
俞岚	2019	耳尖放血疗法治疗高血压性脑出血对效果及住院时间的影响	中西医结合心血管病电子杂志	7	34	81+85	
芦爽	2017	耳尖放血疗法护理高血压性脑出血对效果及住院时间的影响	双足与保健	26	21	118+120	
冯晓东	2015	耳穴放血结合康复训练治疗脑卒中后认知障碍疗效观察	中国临床研究	7	14	49-50	
潘丹	2016	针刺配合放血疗法治疗脑卒中后感觉障碍疗效观察	上海针灸杂志	35	7	782-785	
周黎	2010	刺血疗法对脑梗死致偏身感觉障碍患者脑血流的影响	上海针灸杂志	29	2	83-85	
陈叙波	2017	中冲放血急救干预对于急性脑梗死超早期患者血液流变学水平的影响	中国临床研究	9	35	32-33	No related outcomes
颜世香	2021	三棱针穿刺合并中药熏洗治疗脑卒中偏瘫侧肢体膝关节骨挫伤临床观察	光明中医	36	9	1477-1479	
李俊华	2016	耳尖放血疗法护理高血压性脑出血患者的观察	中国民间疗法	24	11	93-94	
黄汝成	2017	刺血对急性脑梗死患者PDGF、NSE的影响	临床医学工程	24	10	1377-1378	
周智梁	2004	刺血疗法治疗脑血管疾病偏身感觉障碍疗效观察	上海针灸杂志	23	5	6-7	
郝有志	2019	醒脑开窍针法配合法刺络放血治疗脑卒中吞咽障碍40例	世界最新医学信息文摘	19	50	29-30	
陈勇	2010	电针结合背部循经刺络对脑梗死后遗症的康复作用	江苏中医药	42	2	52-53	No skin-puncturing procedure
丁晶	2004	手十二井穴刺络放血对中风初起患者意识状态影响的临床对比观察	中国针灸	24	10	11-14	Single-session bloodletting
郭义	2003	手十二井穴刺络放血对中风患者意识状态、心率等影响的临床观察	天津中医药	20	2	35-37	
N.N. Yu	2021	Wake-Promoting Effect of Bloodletting Puncture at Hand Twelve Jing-Well Points in Acute Stroke Patients: A Multi-center Randomized Controlled Trial	Chinese Journal of Integrative Medicine	27	8	570-577	
Y. Guo	2005	Effect of blood-letting puncture at twelve well-points of hand on consciousness and heart rate in patients with apoplexy	Journal of Traditional Chinese Medicine	25	2	85-89	Use duplicate data
于川	2014	十宣穴放血对急性脑梗死患者神经功能的影响	上海针灸杂志	33	2	111-2	Use unreliable data
