# Supplementary material for: Bloodletting for Acute Stroke Recovery: A Systematic Review and Meta-Analysis
Source: Healthcare (Basel). 2024 Oct 17;12(20):2060. doi: 10.3390/healthcare12202060 (PMC11507497; doi:10.3390/healthcare12202060)
Supplement: Supplementary file 1 [file healthcare-12-02060-s001.zip › Table S4. Subgroup analysis.pdf]

Table S4. Subgroup analysis.

Outcomes		Subgroup / Entire cohort		Stroke type: IS only	Stroke type: The others	Time since onset: ≤ 3 d	Time since onset: > 3 d
NE	NIHSS	No. study	3			2	1
		No. Tx	89			60	29
		No. Con	98	NA		60	28
		I^2 (%)	0			0	NA
		MD	-2.08			-2.25	-1.79
		95%CI	-3.13			-3.58	-3.52
			-1.02			-0.91	-0.06
	CSS	No. study	8	7	1	5	3
		No. Tx	451	426	25	328	123
		No. Con	446	421	25	323	123
		I^2 (%)	0	0	NA	0	0
		MD	-4.15	-4.16	-3.79	-4.32	-3.95
		95%CI	-4.59	-4.60	-6.35	-4.92	-4.59
			-3.71	-3.71	-1.23	-3.72	-3.31
	TER	No. study	14	12	2	7	7
		No. Tx	682	617	65	400	282
		No. Con	637	572	65	396	241
		I^2 (%)	1	15	0	0	46
		RR	1.17	1.17	1.17	1.19	1.17
		95%CI	1.11	1.11	**1.00**	1.12	1.07
			1.22	1.24	**1.37***	1.27	1.28
ADL	BI	No. study	5			3	2
		No. Tx	180			116	64
		No. Con	179	NA		116	63
		I^2 (%)	88			89	0
		SMD	0.53			0.85	0.06
		95%CI	**-0.09**			**0.01**	-0.29
			**1.16***			**1.69#**	0.40
MF	FMA-LE	No. study	3			1	2
		No. Tx	99			30	69
		No. Con	98	NA		30	68
		I^2 (%)	40			NA	69
		MD	3.86			3.77	3.52
		95%CI	2.16			1.37	0.29
			5.56			6.17	6.75

Outcomes			Treatment period: ≤ 1 wk	Treatment period: > 1 wk	Treatment interval: ≤ 2 d/wk	Treatment interval: >2 d/wk	Total session: ≤ 10	Total session: > 10
NE	NIHSS	No. study	1	2	2	1	1	2
		No. Tx	30	59	60	29	30	59
		No. Con	30	58	60	28	30	58
		I^2 (%)	NA	0	0	NA	NA	0
		MD	-2.38	-2.02	-2.25	-1.79	-2.38	-2.02
		95%CI	**-5.04**	-3.17	-3.58	-3.52	**-5.04**	-3.17
			**0.28***	-0.86	-0.91	-0.06	**0.28***	-0.86
	CSS	No. study	1	7	6	2	4	4
		No. Tx	20	431	198	198	303	148
		No. Con	15	431	193	193	298	148
		I^2 (%)	NA	0	0	0	0	0
		MD	-3.60	-4.15	-3.93	-3.93	-4.35	-3.94
		95%CI	**-8.91**	-4.59	-4.55	-4.55	-4.97	-4.56
			**1.71***	-3.71	--3.32	-3.32	-3.74	-3.32
	TER	No. study	1	13	9	5	5	9
		No. Tx	30	652	330	352	345	337
		No. Con	30	607	296	341	331	306
		I^2 (%)	NA	0	0	21	0	28
		RR	1.33	1.16	1.19	1.15	1.20	1.2
		95%CI	1.04	1.11	1.11	1.06	1.11	1.12
			1.72	1.22	1.26	1.24	1.29	1.28
ADL	BI	No. study			4	1	1	4
		No. Tx			151	29	30	150
		No. Con	NA		151	28	30	149
		I^2 (%)			89	NA	NA	90
		SMD			0.65	0.05	0.22	0.61
		95%CI			-0.08	-0.47	-0.29	-0.16
					1.38	0.57	0.72	1.38
MF	FMA-LE	No. study			2	1		
		No. Tx			70	29		
		No. Con	NA		70	28	NA	
		I^2 (%)			0	NA		
		MD			4.54	1.48		
		95%CI			3.26	**-1.90**		
					5.82	**4.86***		

* Unlike the entire cohort analysis, the 95% confidence interval overlaps the null point.

Unlike the entire cohort analysis, the 95% confidence interval does not overlap the null point in the subgroup analysis

Abbreviations. NE: neurological deficit, ADL: activities of daily living function, MF: motor function, National Institute of Health Stroke Scale, CSS: Chinese Stroke Scale, TER: total effective rate, BI: Barthel index, FMA: Fugl-Meyer Assessment, LE: lower extremity, No. study: number of studies, No. Tx: number of participants of treatment group, No. Con: number of participants of control group, MD: mean difference, CI: confidence interval, RR: risk ratio, SMD: standardized mean difference, IS: ischemic stroke, d: day, wk: week, NA: not available.
