# Supplementary material for: Bloodletting for Acute Stroke Recovery: A Systematic Review and Meta-Analysis
Source: Healthcare (Basel). 2024 Oct 17;12(20):2060. doi: 10.3390/healthcare12202060 (PMC11507497; doi:10.3390/healthcare12202060)
Supplement: Supplementary file 1 [file healthcare-12-02060-s001.zip › Table S5. Sensitivity analysis.estimate수정.pdf]

Table S5. Sensitivity analysis using leave-one-out method.

Outcome		Excluded study	Effect size	Lower CI	Upper CI
NE	NIHSS (MD)	None	-2.08	-3.13	-1.02
		LiuDS2008	-2.25	-3.58	-0.91
		ZhangGN2018	-2.02	-3.17	-0.86
		WangZQ2019	-1.97	-3.42	-0.51
	CSS (MD)	None	-4.15	-4.59	-3.71
		HuangJB2002	-4.15	-4.59	-3.71
		LiaoPS2008	-4.15	-4.59	-3.71
		LiuDR2010	-4.18	-4.62	-3.73
		ChengH2013	-4.16	-4.60	-3.71
		RuanJG2014	-4.17	-4.62	-3.73
		QiuJJ2017	-4.22	-4.79	-3.65
		XuYL2020	-4.06	-4.53	-3.59
		ZhaoB2022	-4.10	-4.64	-3.55
	TER (RR)	None	1.17	1.11	1.22
		CuiH2005	1.16	1.11	1.22
		LiuDS2008	1.19	1.13	1.25
		TengAQ2009	1.17	1.11	1.23
		LiuDR2010	1.16	1.11	1.21
		ChengH2013	1.17	1.11	1.23
		ZhangM2013	1.16	1.11	1.22
		RuanJG2014	1.19	1.13	1.25
		ChenXB2015	1.17	1.11	1.23
		QiuJJ2017	1.16	1.10	1.21
		ZhangGN2018	1.16	1.11	1.22
		ShenT2019	1.17	1.11	1.23
		WangZQ2019	1.17	1.11	1.23
		XuYL2020	1.16	1.10	1.21
		ZhaoB2022	1.18	1.12	1.25
ADL	BI (SMD)	None	0.53	-0.09	1.16
		LiuDS2008	0.65	-0.08	1.38
		LiaPS2008	0.61	-0.16	1.38
		RuanJG2014	0.65	-0.09	1.39
		WangZQ2019	0.49	-0.30	1.28
		GuoJY2019	0.25	-0.04	0.53
	TLA (MD)	None	-0.23	-0.6	0.15
		LiuDR2010	-0.38	-1.23	0.47
		ZhangGN2018	-0.19	-0.62	0.24
MF	FMA-UE (MD)	None	12.20	9.67	14.73
		ZhangM2013	12.64	9.87	15.41
		QiuJJ2017	9.97	3.73	16.21
	FMA-LE (MD)	None	3.86	2.16	5.56
		LiuDS2008	4.54	3.26	5.82
		ZhangM2013	3.52	0.29	6.75
		QiuJJ2017	2.59	0.79	5.10
	FMA-Hand (MD)	None	2.79	0.06	5.53
		LiuDS2008	1.76	1.67	1.85
		GuoJY2019	4.8	1.59	7.77
Safety	AE (RR)	None	0.91	0.44	1.91
		LiuDS2008	1.00	0.32	3.10
		WangZQ2019	0.86	0.44	2.25

Abbreviations. NE: neurological deficit, ADL: activities of daily living function, MF: motor function, NIHSS: National Institute of Health Stroke Scale, MD: mean difference, CSS: Chinese Stroke Scale, MD: mean difference, TER: treatment effective rate, RR: risk ratio, BI: Barthel index, SMD: standardized mean difference, TLA: total life ability, FMA: Fugl-Meyer Assessment, UE: upper extremity, LE: lower extremity, AE: adverse event, CI: 95% confidence interval.
